# Hepatitis B and C Co-Infection Are Independent Predictors of Progressive Kidney Disease in HIV-Positive, Antiretroviral-Treated Adults

**DOI:** 10.1371/journal.pone.0040245

**Published:** 2012-07-20

**Authors:** Amanda Mocroft, Jacqueline Neuhaus, Lars Peters, Lene Ryom, Markus Bickel, Daniel Grint, Janak Koirala, Aleksandra Szymczak, Jens Lundgren, Michael J. Ross, Christina M. Wyatt, for the INSIGHT SMART and ESPRIT Study Groups

**Affiliations:** 1 Research Department of Infection and Population Health, University College London Medical School, London, United Kingdom; 2 Department of Biostatistics, University of Minnesota School of Public Health, Minneapolis, Minnesota, United States of America; 3 Copenhagen HIV Programme, Faculty of Health Sciences, University of Copenhagen, Copenhagen, Denmark; 4 Department of Infectious Disease, Internal Medicine, JW Goethe University Clinic, Frankfurt, Germany; 5 Division of Infectious Diseases, Department of Medicine, Southern Illinois University School of Medicine, Springfield, Illinois, United States of America; 6 Department of Infectious Diseases, Liver Diseases and Acquired Immunodeficiencies, Wroclaw Medical University, Wroclaw, Poland; 7 Department of Infectious Diseases, Copenhagen University Hospital/Rigshospitalet, Copenhagen, Denmark; 8 Division of Nephrology, Department of Medicine, Mount Sinai School of Medicine, New York, New York, United States of America; University of Sao Paulo Medical School, Brazil

## Abstract

Chronic kidney disease (CKD) is an important cause of morbidity and mortality in HIV-positive individuals. Hepatitis C (HCV) co-infection has been associated with increased risk of CKD, but prior studies lack information on potential mechanisms. We evaluated the association between HCV or hepatitis B (HBV) co-infection and progressive CKD among 3,441 antiretroviral-treated clinical trial participants. Progressive CKD was defined as the composite of end-stage renal disease, renal death, or significant glomerular filtration rate (eGFR) decline (25% decline to eGFR <60 mL/min/1.73 m^2^ or 25% decline with a baseline <60). Generalized Estimating Equations were used to model the odds of progressive CKD. At baseline, 13.8% and 3.3% of participants were co-infected with HCV and HBV, respectively. Median eGFR was 111, and 3.7% developed progressive CKD. After adjustment, the odds of progressive CKD were increased in participants with HCV (OR 1.72, 95% CI 1.07–2.76) or HBV (OR 2.26, 95% CI 1.15–4.44). Participants with undetectable or low HCV-RNA had similar odds of progressive CKD as HCV seronegative participants, while participants with HCV-RNA >800,000 IU/ml had increased odds (OR 3.07; 95% CI 1.60–5.90). Interleukin-6, hyaluronic acid, and the FIB-4 hepatic fibrosis index were higher among participants who developed progressive CKD, but were no longer associated with progressive CKD after adjustment. Future studies should validate the relationship between HCV viremia and CKD.

**Trial Registration:**

ClinicalTrials.gov NCT00027352; NCT00004978

## Introduction

In 2006, ten years after the widespread introduction of effective combination antiretroviral therapy (cART) for the treatment of human immunodeficiency virus (HIV) infection, the randomized Strategies for Management of Antiretroviral Therapy trial (SMART) established uninterrupted cART with the goal of continuous viral suppression as the standard of care. [1] Although the primary outcome of SMART focused on mortality and opportunistic illness associated with acquired immunodeficiency syndrome (AIDS), the results also highlighted the growing burden of comorbid disease in the cART era. During an average follow-up of 16 months, serious cardiac, liver, and kidney events were more common than serious AIDS-defining events, regardless of treatment assignment. [1].

Chronic kidney disease (CKD) has been associated with increased morbidity and mortality in HIV-positive individuals receiving cART. [2]–[3] In addition to traditional CKD risk factors such as diabetes and hypertension, co-infection with hepatitis C virus (HCV) has been suggested as a possible risk factor for CKD in HIV-positive individuals. [4] Although there are conflicting data on the relationship between HCV infection and CKD in the general population, meta-analysis of published studies in HIV-positive populations supports an association between HIV-HCV co-infection and increased risk of CKD. [5] The relationship between hepatitis B virus (HBV) infection and CKD has not been as extensively studied, although cross-sectional studies have not demonstrated an association between HBV mono-infection and prevalent CKD. [6]–[7].

Both HBV and HCV have been implicated in the pathogenesis of specific immune complex kidney diseases in the general population and in HIV-positive individuals,[8]–[11] and clinically silent immune complex kidney disease has been observed in HCV mono-infected patients with end-stage liver disease. [12] End-stage liver disease has also been associated with increased risk of overt CKD in HCV-infected individuals, although data on the relative contribution of immune complex disease and hepatorenal syndrome were not available. [13] Previous studies have not investigated other potential mediators of the relationship between viral hepatitis and CKD, including earlier stages of hepatic fibrosis and liver dysfunction, increased levels of systemic inflammation, or nephrotoxic effects of antiviral therapy for HBV or HCV. In addition, the majority of prior studies defined viral hepatitis co-infection by serology alone, and did not report data on HBV DNA, HCV RNA, or HCV genotype as potential mediators or effectors of the relationship between viral hepatitis and CKD.

We evaluated the association between viral hepatitis co-infection and progressive CKD among 3,441 cART-treated participants enrolled in two large international HIV treatment trials, with the goal of identifying potential mediators of the relationship.

## Methods

### Study Population

The study designs of SMART and the Evaluation of Subcutaneous Proleukin in a Randomized International Trial (ESPRIT) have been described previously. [1], [14] Briefly, SMART randomized 5,472 HIV-positive adults with CD4 cell count (CD4) >350 cells/mm^3^ to receive uninterrupted cART with the goal of viral suppression *versus* episodic cART guided by CD4. SMART was stopped early because of a safety risk in the episodic therapy arm. [1] ESPRIT randomized 4,111 HIV-positive adults with CD4>300 cells/mm^3^ to receive cART alone or in combination with subcutaneous interleukin-2. ESPRIT failed to demonstrate a clinical benefit of interleukin-2 despite an increase in CD4. [14] For consistency with standards of care, the current analysis included only participants randomized to the viral suppression arm of SMART and the control arm of ESPRIT. Baseline was defined as the date of randomization into SMART or ESPRIT. Eligible participants with plasma specimens available for centralized measurement of creatinine at baseline and at least one subsequent study visit were included in this analysis.

### Definition of Study Endpoints and Covariates

Plasma specimens were collected in EDTA tubes, aliquoted, and shipped frozen to a central repository. Available specimens from the randomization (“baseline”), 12-month, and subsequent annual visits were retrieved for centralized creatinine testing using an isotope dilution mass spectrometry (IDMS)-traceable enzymatic assay (Roche Creatinine Plus in SMART and Diazyme Liquid Reagents Creatinine Assay in ESPRIT). The eGFR was calculated from centralized creatinine values using the Chronic Kidney Disease Epidemiology Consortium (CKD-EPI) equation. [15] In a sensitivity analysis, we explored the impact of adjusting eGFR in Asian participants using a correction coefficient of 0.813, as suggested for Japanese individuals, [16] or of 1.052, which was shown to improve the bias of the CKD-EPI equation among Chinese individuals. [17].

For the purposes of this analysis, progressive CKD was defined as the composite of end-stage renal disease (ESRD), renal death, or significant decline in eGFR (25% decline in eGFR to a level ≤60 mL/min/1.73 m^2^ in participants with a baseline eGFR >60 mL/min/1.73 m^2^ or a 25% decline in eGFR for those with a baseline eGFR <60 mL/min/1.73 m^2^). To address the potential misclassification of acute kidney injury in participants with a 25% decline in eGFR based on a single follow-up creatinine value, we performed a sensitivity analysis requiring a confirmed 25% decline in eGFR based on at least 2 consecutive values.

Hepatitis co-infection was defined serologically at baseline, based on the detection of HBV surface antigen (HBsAg) or anti-HCV antibody. Additional testing was performed in participants with serologic evidence of HBV or HCV co-infection, including HBV-DNA or HCV-RNA and HCV genotype, respectively. Relevant comorbid conditions were defined at baseline. Hypertension, diabetes, and hyperlipidemia were defined by the self-reported use of medications to treat these conditions. Cardiovascular disease was defined by self-reported medical treatment or a history of coronary revascularization, myocardial infarction, or cerebrovascular accident prior to baseline. Plasma hyaluronic acid was measured as a circulating marker of hepatic fibrosis in co-infected participants, [18] and the FIB-4 and APRI fibrosis indices were calculated for all participants with available data at baseline.[19]–[20] The systemic inflammatory markers IL-6 and hsCRP were also measured at baseline in approximately 70% of participants, independent of hepatitis status.

### Statistical Methods

Descriptive statistics were used to compare baseline characteristics between participants enrolled in SMART and ESPRIT, and between participants who did or did not develop progressive CKD. A descriptive analysis was also performed to compare the proportion of participants with progressive CKD at yearly intervals. The odds of progressive CKD were investigated using Generalised Estimating Equations, using binomial regression and adjusting for repeated measurements per person. Participants were included in analyses until the development of progressive CKD or the last eGFR measurement. In addition to HBV or HCV co-infection, other potential explanatory variables included age, sex, race, HIV exposure category, history of AIDS-defining illness, presence of other relevant comorbid conditions, HIV-RNA, CD4, CD4 nadir, body mass index (BMI), eGFR, and the use of cART both prior to and at randomization into the parent trial. Any explanatory variables with p<0.1 in univariate analyses were included in multivariate analyses. Excluded variables were tested in the final model to determine if their inclusion improved the model fit. Separate sensitivity analyses were performed including time-updated (“on-treatment”) variables for selected antiretroviral agents, and including only participants with progressive CKD as defined by a clinical CKD event or a confirmed decline in eGFR based on two consecutive measures.

Further analyses focused on the role of baseline HBV or HCV viremia and HCV genotype as explanatory variables; HBV and HCV viremia were also explored as time-updated variables. Exploratory analyses using the same methods investigated the role of baseline hyaluronic acid, APRI, FIB-4, IL6 and hsCRP. Separate multivariate models were run for each of these markers to minimize the impact of missing data; as a result, the presented results were not mutually adjusted for the other markers. Multivariate models were adjusted for the same factors that were found to be of importance in the main analysis.

## Results

A total of 4,792 participants were enrolled in the standard therapy arms of SMART and ESPRIT. [1], [14] After excluding 971 participants with no centralized plasma creatinine at enrollment (“baseline”) and an additional 380 participants with no subsequent centralized creatinine, 3,441 participants (72%) were included in the current analysis (2,054 from SMART and 1,387 from ESPRIT). Compared to those excluded, included participants were less likely to report their race as white and more likely to be enrolled in ESPRIT, had lower CD4+ T-cell count (CD4) nadirs, were randomized later, and were more likely to be on lipid-lowering therapy. The prevalence of serologically defined HBV and HCV co-infection was similar in excluded and included participants.

A total of 11,050 follow-up plasma creatinine values were available to calculate estimated glomerular filtration rate (eGFR), a median of 3 (interquartile range [IQR] 2–5) per participant. There were more creatinine measurements available in participants from ESPRIT compared to SMART (median 5 versus 2 respectively, p<0.0001), consistent with the longer follow-up period, and in those who developed progressive CKD compared to those who did not (median 5 versus 3 respectively, p<0.001). The median time between measurements was 1 year (IQR 1.0–1.1), with no difference between trials (p = 0.59) and in those who did and did not develop CKD (p = 0.22). Other characteristics associated with more available creatinine measurements included lower baseline CD4, higher nadir CD4, prior AIDS diagnosis, older age, and non-white race.

### Baseline Characteristics of the Study Population

Table 1 summarizes the characteristics of the study population at randomization into SMART or ESPRIT, comparing those who did and did not develop progressive CKD. There were a total of 114 participants (3.3%) with serologic evidence of HBV co-infection as defined by HBV surface antigen (HBsAg) and 473 (13.8%) participants with serologic evidence of HCV co-infection as defined by anti-HCV antibody. The prevalence of HBV co-infection was higher in ESPRIT participants (5.3% *versus* 2.0%), while the prevalence of HCV co-infection was similar across trials. At baseline, 2,999 (87.2%) participants had an eGFR >90 mL/min/1.73 m^2^, and 57 participants (1.7%) had an eGFR <60 mL/min/1.73 m^2^. The proportions with normal and decreased eGFR were similar across trials. Participants in SMART were more likely to report their race as black, more likely to report comorbid diabetes, hypertension, and cardiovascular disease, and less likely to have suppressed HIV-RNA.

**Table 1:** Baseline characteristics of study participants, stratified by the development of progressive chronic kidney disease (CKD).

		All(n = 3,441)		No progressive CKD(n = 3,314)		Progressive CKD(n = 127)		p
Median age	Years	43	36,47	42	36,39	48	39,55	<0.0001
Male sex		2638	76.7	2544	76.8	94	74.0	0.47
Race	Black	693	20.1	663	20.0	30	23.6	<0.0001
	Asian	206	6.0	185	5.6	21	16.5	–
	White	2280	66.3	2211	66.7	69	54.4	–
	Other	262	7.6	255	7.7	7	5.5	–
Exposure category	Intravenous drug use	331	9.6	315	9.5	17	13.4	0.14
	Heterosexual contact	1387	40.3	1331	40.2	56	44.1	0.38
	Same sex contact	1812	52.7	1745	53.0	56	44.1	0.049
Median CD4	cells/mm^3^	524	414, 700	526	415, 702	457	390, 570	<0.0001
Median nadir CD4	cells/mm^3^	223	114, 330	226	117, 333	153	87, 262	<0.0001
HIV RNA	<500 copies/mL^1^	2626	76.5	2533	76.6	93	73.2	0.38
History of AIDS		906	26.3	862	26.0	44	34.7	0.030
Antiretroviral naïve		786	22.8	765	23.1	21	16.5	0.085
Specific antiretrovirals								
Atazanavir use	At randomization	128	3.7	128	3.9	0	0	0.024
	Prior history	29	0.8	28	0.8	1	0.8	0.94
Indinavir use	At randomization	304	8.8	283	8.5	21	16.5	0.0018
	Prior history	980	28.5	942	28.4	38	29.9	0.71
Ritonavir use	At randomization	413	12.0	395	11.9	18	14.2	0.44
	Prior history	603	17.5	578	17.4	25	19.7	0.51
Tenofovir use	At randomization	419	12.2	411	12.4	8	6.3	0.039
	Prior history	84	2.4	82	2.5	2	1.6	0.52
Hepatitis C virus	Antibody positive+^2^	473	13.8	448	13.5	25	19.7	0.049
	RNA positive^3^	363	77.1	342	76.7	21	84.0	0.40
	RNA >800,000	151	32.1	139	31.1	12	48.0	0.079
	Genotype 1^3^	258	73.1	240	72.3	18	85.7	0.18
Hepatitis B virus	Surface antigen positive^1^	114	3.3	100	3.0	14	11.0	<0.0001
	DNA positive	70	61.4	62	62.0	8	57.1	0.73
Median eGFR	ml/min/1.73 m^2^	111	100, 121	112	101, 121	91	79, 106	<0.0001
Median BMI	kg/m^2^	24	22, 27	24	22, 27	24	22, 27	0.19
Comorbid conditions	Diabetes mellitus	177	5.1	159	4.8	18	14.2	<0.0001
	Antihypertensive therapy	447	13.0	415	12.5	32	25.2	<0.0001
	Lipid-lowering therapy	501	14.6	475	14.3	26	20.5	0.054
	Cardiovascular disease	114	3.3	109	3.3	5	3.9	0.69

Baseline was defined at randomization into the parent trial (SMART or ESPRIT).

Categorical variables are presented as N (%) and continuous variables presented as median (interquartile range).

1 Data on HIV RNA and Hepatitis B surface antigen were available for 3435 participants (99.8%).

2 Data on Hepatitis C antibody status were available for 3436 participants (99.8%).

3 Among 473 participants with positive Hepatitis C antibody, RNA viral load was available for 471 (99.6%) and genotype was available for 353 (74.6%).

eGFR, estimated GFR calculated using the CKD-EPI formula; BMI, body mass index.

### Development of Progressive CKD

**Figure 1:**
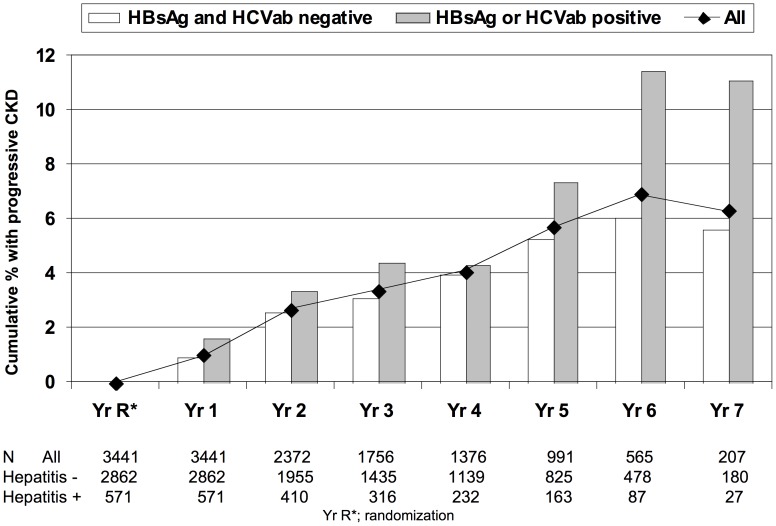
Cumulative proportion of participants with progressive kidney disease.

A total of 127 participants (3.7%) developed progressive CKD. The majority of events occurred in participants with a baseline eGFR >60 mL/min/1.73 m^2^ (n = 119). Nearly all events were defined by eGFR decline (n = 125), although three of these participants subsequently developed ESRD during follow-up. Two additional participants developed a clinical event without a documented decline in eGFR of at least 25% (one case of ESRD and one renal death). Forty-two events (2.0%) occurred in SMART participants and 85 events (6.1%) occurred in ESPRIT participants (p<0.0001). For descriptive purposes, Figure 1 shows the cumulative proportion of participants with progressive CKD each year after baseline, overall and stratified according to the presence of HBV or HCV co-infection. The proportion with progressive CKD was higher among participants with HBV or HCV co-infection at all time points. In univariate analysis, both HBV and HCV co-infection were associated with increased odds of progressive CKD (Table 2). No CKD events occurred in the small number of participants co-infected with both HBV and HCV (n = 16).

**Table 2:** Baseline characteristics associated with progressive CKD in univariate and multivariate analysis.

	Univariate		Multivariate	
	Odds Ratio	95% CI	Odds Ratio	95% CI
Hepatitis B surface antigen positive	3.28	1.90–5.67	2.26	1.15–4.44
Hepatitis C antibody positive	1.58	1.02–2.44	1.72	1.07–2.76
Age, per 10 years	1.69	1.41–2.02	1.36	1.10–1.68
Race				
Black	1.59	1.04–2.45	2.07	1.23–3.49
Asian	2.43	1.49–3.97	2.33	1.19–4.55
Other	0.97	0.45–2.11	1.27	0.57–2.83
Same sex exposure	0.70	0.49–0.99	0.76	0.51–1.13
CD4, per 100 cells/mm^3^	0.88	0.80–0.97	1.00	0.90–1.11
Nadir CD4, per 100 cells/mm^3^	0.79	0.70–0.89	0.84	0.72–0.98
History of AIDS	1.51	1.05–2.17	1.13	0.74–1.72
Antiretroviral naïve	0.65	0.41–1.04	0.80	0.49–1.30
Current or previous indinavir use	1.68	1.05–2.70	0.76	0.43–1.34
Antihypertensive therapy	2.62	1.75–3.90	1.83	1.14–2.94
Lipid-lowering therapy	1.61	1.04–2.47	1.28	0.80–2.05
eGFR, per 5 ml/min/1.73 m^2^	0.82	0.80–0.85	0.85	0.82–0.87
Enrolled in ESPRIT	1.77	1.22–2.56	1.77	0.90–3.49
Date of randomization, per year	0.80	0.71–0.90	0.81	0.67–0.98

In multivariate analysis adjusting for baseline characteristics, the odds of developing progressive CKD were significantly increased in participants with HBV (adjusted OR 2.26, 95% CI 1.15–4.44) or HCV co-infection (adjusted OR 1.72, 95% CI 1.07–2.76). Other factors associated with increased odds of progressive CKD included black or Asian race, older age, and self-reported use of antihypertensive medications. Higher baseline eGFR and higher CD4 nadir were associated with lower odds of developing progressive CKD, as was a later date of randomization into the parent trial.

### Sensitivity Analyses

In sensitivity analysis, the inclusion of time-updated “on-treatment” variables for potentially nephrotoxic antiretroviral agents (atazanavir, indinavir, boosted lopinavir, and tenofovir) and agents with dual activity against HIV and HBV (lamivudine and emtricitabine) did not significantly affect the relationship between progressive CKD and HBV (adjusted OR 2.20, 95% CI 1.13–2.89) or HCV co-infection (adjusted OR 1.77, 95% CI 1.08–2.89). In a separate sensitivity analysis including 2,659 participants with at least 2 centralized creatinine values during follow-up, 30 participants (1.1%) developed progressive CKD as defined by a clinical event or a decline in eGFR confirmed on two consecutive measures. In adjusted analysis, the odds ratios associated with hepatitis virus co-infection were similar to the primary analysis but no longer reached statistical significance (HBV co-infection 1.94, 95% CI 0.46–8.18; HCV co-infection 1.73, 95% CI 0.61–4.89). Other relationships were consistent with the results of the primary analysis (data not shown). In a final sensitivity analysis, we explored the impact of adjusting eGFR in Asian participants using recommended correction coefficients for Japanese and Chinese populations.[16]–[17] While these adjustments impacted the number of CKD events in Asian participants, the relationships between HBV, HCV, Asian race, and progressive CKD were similar to the primary analysis.

### Planned Subgroup Analysis of Participants with HBV or HCV Co-infection

**Figure 2:**
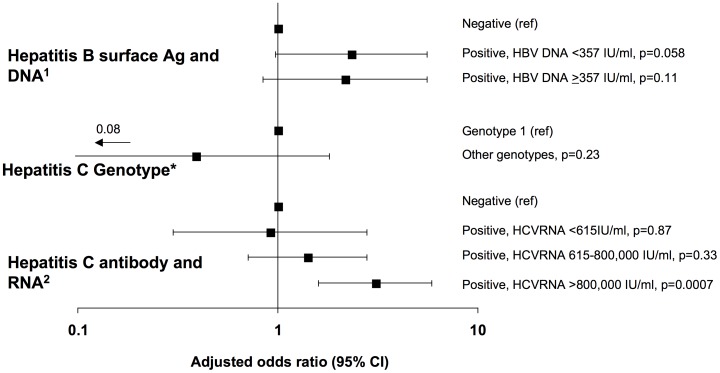
Association of Hepatitis B and C viremia and Hepatitis C genotype with progressive kidney disease. Final models were adjusted for trial, age, race, infection through same sex exposure, prior AIDS diagnosis, CD4, CD4 nadir, previous exposure to antiretrovirals, previous exposure to indinavir, treatment with antihypertensives, treatment with lipid lowering therapy, estimated glomerular filtration rate, and year of randomization, all measured at randomization into the parent trial. As appropriate, analyses were also adjusted for ^1^Hepatitis C (HCV) antibody status or ^2^ Hepatitis B surface antigen (HBsAg) status. Participants with positive HBsAg were further stratified by the presence or absence of detectable HBV DNA, with a cutoff of <357 IU/ml. The number of patients (events) for HBsAg negative, positive with HBV DNA <357 IU/mL, and positive with HBV DNA ≥357 IU/ml were 3321 (113), 44 (6), and 70 (8), respectively. Participants with positive HCV antibody were further stratified into those with undetectable HCV RNA, low HCV RNA (≤800,000 IU/ml), and high HCV RNA (>800,000 IU/ml). The number of patients (events) for HCV antibody negative, positive with undetectable HCV RNA, low HCV RNA, and high HCV RNA were 2963 (102), 108 (4), 212 (9), and 151 (12), respectively. The analysis of HCV genotype was limited to participants with positive HCV antibody and known HCV genotype, stratified as genotype 1 (258 patients, 18 events) or other (95 patients, 3 events).

Participants with serologic evidence of HBV co-infection were further stratified by the presence or absence of detectable HBV DNA at baseline, with a cutoff of <357 IU/ml (Figure 2). Among 70 participants with detectable HBV DNA, the median HBV DNA was 7.3 (IQR 5.3–7.3) log_10_ copies/mL. After adjustment for the variables in Table 2, participants with serologic evidence of HBV co-infection had similar odds of developing progressive CKD regardless of whether HBV DNA was undetectable (adjusted OR 2.16; 95% CI 0.84–5.55) or detectable at baseline (adjusted OR 2.33; 95% CI 0.97–5.58), but this failed to reach statistical significance in these smaller subgroups. Because of the small number of participants with HBV co-infection, it was not possible to further stratify viremic participants or to explore the role of HBV rebound during follow-up.

**Figure 3:**
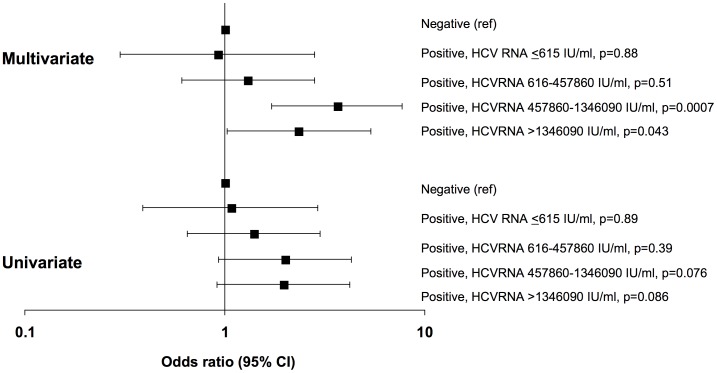
Association of Hepatitis C viremia with progressive kidney disease. Hepatitis C (HCV)-RNA was further stratified by quartiles to determine whether there is clinically useful threshold HCV-RNA level associated with increased risk of progressive kidney disease. The number of patients (events) included for HCV antibody negative and for increasing quartiles of HCV-RNA were 2963 (102), 108 (4), 154 (7), 102 (7), and 107 (7), respectively. Final models were adjusted for trial, age, race, infection through same sex exposure, prior AIDS diagnosis, CD4, CD4 nadir, previous exposure to antiretrovirals, previous exposure to indinavir, treatment with antihypertensives, treatment with lipid lowering therapy, estimated glomerular filtration rate, year of randomization, and Hepatitis B surface antigen status, all measured at randomization into the parent trial.

Participants with serologic evidence of HCV co-infection were further stratified into those with undetectable HCV RNA, low HCV RNA (≤800,000 IU/ml), and high HCV RNA (>800,000 IU/ml). After adjustment, participants with undetectable HCV RNA (adjusted OR 0.91, 95% CI 0.30–2.79) or with low HCV RNA (adjusted OR 1.41; 95% CI 0.71–2.79) had similar odds of developing progressive CKD as those with a negative HCV antibody result, while participants with high HCV RNA had significantly increased odds (adjusted OR 3.07; 95% CI 1.60–5.90). Among those with HCV co-infection, the test for trend moving from those with undetectable HCV RNA to those with high HCV RNA was marginally statistically significant (p = 0.057). Similar results were seen when RNA levels were stratified by the median value (585,000 IU/ml) among viremic participants (data not shown). Further stratification of HCV RNA levels by quartiles suggested a gradual increase in risk of progressive CKD associated with increasing HCV RNA levels. The test for trend was again marginally statistically significant (p = 0.066), and the small number of events in each stratum meant that the confidence intervals for individual strata were wide (Figure 3). Among 353 participants with known HCV genotype, there were no differences in the odds of developing progressive CKD in those with genotype 1 versus other genotypes (Figure 2).

Circulating levels of hyaluronic acid, as well as the calculated FIB-4 index of hepatic fibrosis, were significantly higher at baseline among participants who subsequently developed progressive CKD, while there was no significant difference in the aspartate aminotransferase platelet ratio index (APRI). None of the markers of hepatic fibrosis remained significantly associated with progressive CKD after adjustment for other important variables (Table 3). The small number of participants with significant elevations in these markers precluded consideration of clinically relevant cutoff values. As previously reported, the systemic inflammatory markers high sensitivity C-reactive protein (hsCRP) and interleukin-6 (IL-6) were not significantly associated with progressive CKD in this population after adjustment for other variables (Abstract # O-271, Conference on Retroviruses and Opportunistic Infections 2012). The relationships between HBV and HCV co-infection and progressive CKD were not significantly affected by inclusion of any individual marker of hepatic fibrosis or systemic inflammation in separate multivariate models (data not shown).

**Table 3:** Markers of hepatic fibrosis as proposed mediators of progressive chronic kidney disease (CKD).

	No progressive CKD	Progressive CKD			Univariate		Multivariate
	Median (IQR)	Median (IQR)		OR	95% CI	OR	95% CI
Hyaluronic acid^1^	25 (15, 44)	36 (17, 72)	Per 20 higher	1.05	1.02, 1.08	1.00	0.98, 1.03
APRI^2^	0.27 (0.19, 0.40)	0.30 (0.21, 0.49)	Per 1 higher	1.02	0.95, 1.11	1.04	0.89, 1.21
FIB4^3^	0.86 (0.63, 1.19)	1.08 (0.74, 1.51)	Per 1 higher	1.02	0.99, 1.06	0.97	0.71, 1.33

Data available for^ 1^n = 827,

2 n = 1421,

3 n = 1268.

The number of participants who developed progressive kidney disease was ^1^n = 42,

2 n = 73,

3 n = 63.

APRI, aspartate aminotransferase platelet ratio index.

### Post-hoc Subgroup Analyses

Because of the unexpected relationship between HBV co-infection and progressive CKD, this relationship was further investigated after stratification by race and by clinical trial. After adjustment, HBV co-infection was associated with a greater than 6-fold increased odds of CKD in SMART and a 1.5-fold increased odds in ESPRIT, but there was no evidence that the adjusted OR was significantly different between the trials (p = 0.12 for interaction). When stratified by race, the odds of progressive CKD associated with HBV co-infection were highest in black participants (OR 14.54, 95% CI 4.16–50.85) and lowest in Asian participants (adjusted OR 1.26, 95% CI 0.36–4.47), although this difference was not statistically significant (p = 0.67 for interaction).

We also considered the use of antiviral agents with dual activity against HIV and HBV, as well as the use of antiviral agents for the treatment of HBV. Among 114 participants with HBV co-infection, 12 (10.5%) were taking tenofovir, 76 (66.7%) were taking lamivudine, and none were taking emtricitabine at baseline. One additional participant reported prior use of tenofovir, and 31 reported prior use of lamivudine. There were no differences between participants with and without HBV co-infection in the use of these agents at or prior to baseline (p>0.05 all comparisons). As described above, the inclusion of “on-treatment” variables for these agents did not affect the relationship between HBV and progressive CKD in sensitivity analysis. Among 74 ESPRIT participants with HBV, none reported the use of adefovir at baseline; data on the use of adefovir were not routinely collected in SMART. The anti-HBV agents entecavir and telbivudine were not approved for clinical use during enrollment in the parent trials.

## Discussion

In this analysis of data and specimens from two large randomized HIV treatment trials, co-infection with either HCV or HBV was independently associated with progressive CKD among HIV-positive adults receiving cART. After adjusting for other important characteristics, the relationship between viral hepatitis co-infection and CKD did not appear to be mediated by mild hepatic fibrosis or by systemic inflammation. Increasing plasma HCV RNA, but not HBV DNA, was an independent predictor of progressive CKD. These results support current guidelines that consider HCV co-infection a risk factor for CKD. [4] The observed relationship between HCV viremia and CKD is also consistent with a recent report from a large European HIV cohort. [21] If confirmed in future studies, the relationship between HCV RNA and CKD may provide an additional impetus for antiviral therapy as newer agents become available for the treatment of HCV co-infection.

Although we observed an unexpected relationship between serologic evidence of HBV co-infection and progressive CKD, this relationship did not appear to require active HBV replication at baseline. More refined stratification of baseline HBV DNA and consideration of viral rebound during follow-up were limited by the small number of patients with active HBV. In addition, HBV e antigen (HBeAg) and quantitative HBsAg were not measured, and HBV DNA was only measured in participants with serologic evidence of HBV infection. Data on the use of adefovir for the treatment of HBV were not collected in SMART, so it was not possible to exclude an effect of this potentially nephrotoxic antiviral agent. Adjustment for the use of tenofovir, which may have been used preferentially in the setting of HBV co-infection, did not change our findings.

Suppression of HBV replication with interferon or lamivudine has been associated with remission of kidney disease in some, but not all, cases of HBV-related immune complex kidney disease.[22]–[23] The pathogenesis of HBV-related kidney disease is hypothesized to involve the deposition of HBeAg in glomerular capillaries. [8] Although we were unable to explore this hypothesis in the current study, it is possible that circulating HBV antigens may continue to deposit in the kidney, or that previously deposited antigens may continue to trigger an immune response in the kidney, even in the absence of active HBV replication. This effect may be magnified in patients with HIV co-infection, who are less likely to clear HBV antigens even when HBV DNA is suppressed. [24] Future studies should collect data on proteinuria, hematuria, and circulating markers of immune complex disease [8]–[11] in order to evaluate this hypothesis.

In addition to the novel relationships observed in this study, progressive CKD was also associated with traditional CKD risk factors. Other characteristics that were independently associated with progressive CKD included older age, black race, and hypertension, as well as lower baseline eGFR and lower nadir CD4. These findings are consistent with expert guidelines that recommend increased frequency of CKD screening in individuals with these risk factors. [4] Diabetes was rare in our population, and did not remain independently associated with progressive CKD in multivariate analysis. Female sex and body mass index (BMI) at baseline were not associated with progressive CKD in our population, in contrast to some prior studies in HIV-positive populations.[25]–[26] Of note, very low BMI was rare and women made up less than a quarter of our study population. While Asian race has not been associated with CKD in the setting of HIV infection, Asian nations report some of the highest incidence rates of ESRD in the general population. [27].

Strengths of the current analysis include a large patient population treated according to the standard of care for HIV infection, centralized measurement of serum creatinine, adjustment for markers of HIV disease severity, and inclusion of data on HBV and HCV viremia. Despite these strengths, several limitations should be considered when interpreting the results of this study. Most importantly, this was a secondary analysis of data from randomized clinical trials designed to evaluate non-renal outcomes. Nonetheless, all clinical events were reviewed centrally, and the majority of clinically relevant CKD events were captured based on eGFR decline. It is more likely that we misclassified acute events using the eGFR criteria; however, we obtained similar results in a sensitivity analysis that required confirmation of eGFR decline on two consecutive measures. Second, we had incomplete data on markers of hepatic fibrosis and systemic inflammation. Although we did not observe a relationship between these markers and progressive CKD, clinically significant hepatic fibrosis was rare in this population of clinical trial participants. Because of the small number of participants with elevated markers of hepatic fibrosis, we were also unable to dichotomize these markers at clinically relevant cutoffs. [13] Third, we were unable to fully adjust for cumulative exposure to tenofovir and other potentially nephrotoxic antiretroviral agents, [28] although the inclusion of time-updated “on treatment” variables for these agents did not affect the relationship between HBV or HCV co-infection and progressive CKD. This may have lead to some residual confounding, particularly in the observed association between HBV co-infection and progressive CKD. Of note, tenofovir and atazanavir were infrequently used at the time of enrollment in the parent studies, and this study was not powered to exclude an association of these agents with CKD. Fourth, we were unable to account for the duration and type of injection drug use as potential confounders of the relationship between HBV or HCV co-infection and progressive CKD. Injection drug use was reported as a risk factor for HIV exposure in fewer than 10% of participants, and exposure history was included in our adjusted analyses. Finally, we were unable to adjust for proteinuria and blood pressure, as these data were not collected in SMART and ESPRIT. Proteinuria is a strong predictor of CKD progression in HIV-positive individuals, [29] and HCV mono-infection has been associated with increased prevalence of proteinuria. [30] Hypertension, as defined by the use of antihypertensive medication at baseline, was associated with progressive CKD in our population. Unfortunately, data on the use of specific antihypertensive agents were not rigorously collected in these HIV treatment trials, and we were unable to consider potential risks or benefits associated with specific agents or classes. [4].

In summary, in this large cohort of HIV-positive clinical trial participants, co-infection with either HBV or HCV was independently associated with progressive CKD. The observed relationship did not appear to be mediated by early hepatic fibrosis or by increased systemic inflammation in co-infected individuals, although we were unable to exclude a role for more advanced liver disease in this relatively healthy population. Active HCV replication was an independent predictor of progressive CKD, and future trials of direct acting antivirals for HCV should consider the impact of successful antiviral treatment on the risk of CKD in co-infected individuals. Future studies are needed to confirm the observed relationship between HBV co-infection and progressive CKD.
